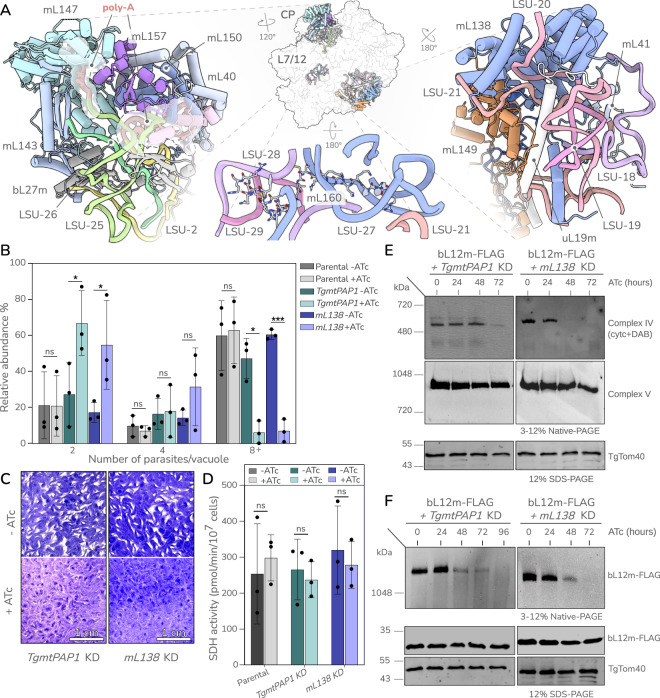# Publisher Correction: Numerous rRNA molecules form the apicomplexan mitoribosome via repurposed protein and RNA elements

**DOI:** 10.1038/s41467-025-57597-w

**Published:** 2025-03-06

**Authors:** Shikha Shikha, Victor Tobiasson, Mariana Ferreira Silva, Jana Ovciarikova, Dario Beraldi, Alexander Mühleip, Lilach Sheiner

**Affiliations:** 1 School of Infection and Immunity, University of Glasgow, Glasgow, Scotland UK; 2 Glasgow Centre for Parasitology, University of Glasgow, Glasgow, Scotland UK; 3 National Center for Biotechnology Information, National Library of Medicine, Bethesda, MD USA; 4 Institute of Biotechnology, Helsinki Institute of Life Science HiLIFE, University of Helsinki, Helsinki, Finland

**Keywords:** Mitochondria, Ribosome, Parasite biology, Cryoelectron microscopy

Correction to: *Nature Communications* 10.1038/s41467-025-56057-9, published online 18 January 2025

In this article the panels for *mL138* KD were interchanged in Fig. 3C; the figure should have appeared as shown below. The original article has been corrected.

**Figure:**