# Supplementary material for: Numerous rRNA molecules form the apicomplexan mitoribosome via repurposed protein and RNA elements
Source: Nat Commun. 2025 Jan 18;16:817. doi: 10.1038/s41467-025-56057-9 (PMC11742926; doi:10.1038/s41467-025-56057-9)
Supplement: Supplementary file 1 — Supplementary Information [file 41467_2025_56057_MOESM1_ESM.pdf]

Numerous rRNA molecules form the Apicomplexan mitoribosome via repurposed protein and RNA elements

Shikha Shikha[1,2*], Victor Tobiasson[3*], Mariana F Silva[1,2], Jana Ovciarikova[1,2], Dario Beraldi[1], Alexander Mühleip[1,2,4,±] and Lilach Sheiner[1,2±]

Supplementary Information:

Supplementary Table 1. Cryo-EM data collection, refinement and validation statistics

Supplementary Fig 1: Generating FLAG tagged bL12m cell line and subsequent immunoprecipitation (IP).
Supplementary Fig 2: CryoEM of the *T. gondii* mitochondrial ribosome.
Supplementary Fig 3: Conservation of new mitoribosomal proteins across myzozoans or apicomplexans, and an apicomplexan structurally conserved insertion in uL23m.
Supplementary Fig 4: “Twin-elements” present on the *T. gondii* mitoribosome.
Supplementary Fig 5: Genomic architecture of rRNA fragments and sequence reuse.
Supplementary Fig 6: 2D diagram of modelled *T. gondii* rRNA.
Supplementary Fig 7: rRNA fragmentation comparison.
Supplementary Fig 8: CryoEM density examples.
Supplementary Fig 9: Poly-A tails integrated into the mitoribosome RNA core and reduced upon *TgmtPAP1* knockdown.
Supplementary Fig 10: Generation and validation of *mL138* and *TgmtPAP1* knockdown mutant.
Supplementary Fig 11: Generation, validation and mitoribosome defect of *mL161* and *mL157* knockdowns.
Supplementary Fig 12: Comparison between the mRNA channels in the Toxoplasma mitoribosome and the bacterial ribosome.
Supplementary Fig 13: Charge properties of the ribosomal exit tunnel.
Supplementary Fig 14: Four ApiAP2 members form integral part of the *T. gondii* mitoribosome .

Data collection	*T. gondii* mtLSU	*T. gondii* mtSSU
**Microscope**	Titan Krios	
**Voltage (kV)**	300	
**Camera**	K3 Quantum	
**Magnification**	165,000	
**Exposure (e^{-}/Å^{2})**	35	
**Pixel size (Å)**	0.83	
**Defocus range (mm)**	0.4-1.4	
**Movies collected**	30,431	
**Frames / movie**	40	
	**Data processing**	
**Initial particles**	2,919,818	
**Final particles**	375,745	
**Symmetry**	C1	C1
**Map resolution (Å)**	2.24	2.52
**- FSC threshold**	0.143	0.143
**B-factor sharpening**	31.6	40.8
**EMDB ID**	EMD-52551	EMD-52348
	**Model refinement statistics**	
**CC (map/model)**	0.72	0.74
**Resolution (map/model, Å)**	2.5	3.0
**- FSC threshold**	0.5	0.5
**Atoms (not H)**	324,059	244,896
**Residues**	15,972	12,979
**Nucleotides**	1955	1101
**ADP (B factors)**		
- **Protein**	44.71	54.69
- **Nucleotide**	21.42	46.21
- **Ligands**	50.08	40.54
**Q-Score (MapQ, sigma 0.6)**	0.64	0.54
**Rotamer outliers (%)**	0.20	0.12
**Ramachandran:**		
- **Outliers**	0.01	0.04
- **Allowed**	2.48	2.52
- **Favoured**	97.50	97.44
**Clash score**	5.06	6.02
**MolProbity score**	1.37	1.44
- **Bonds (Å)**	0.006	0.006
- **Angles (°)**	0.646	0.714
**PDB ID**	9I05	9HQV

Supplementary Table 1. Cryo-EM data collection, refinement and validation statistics

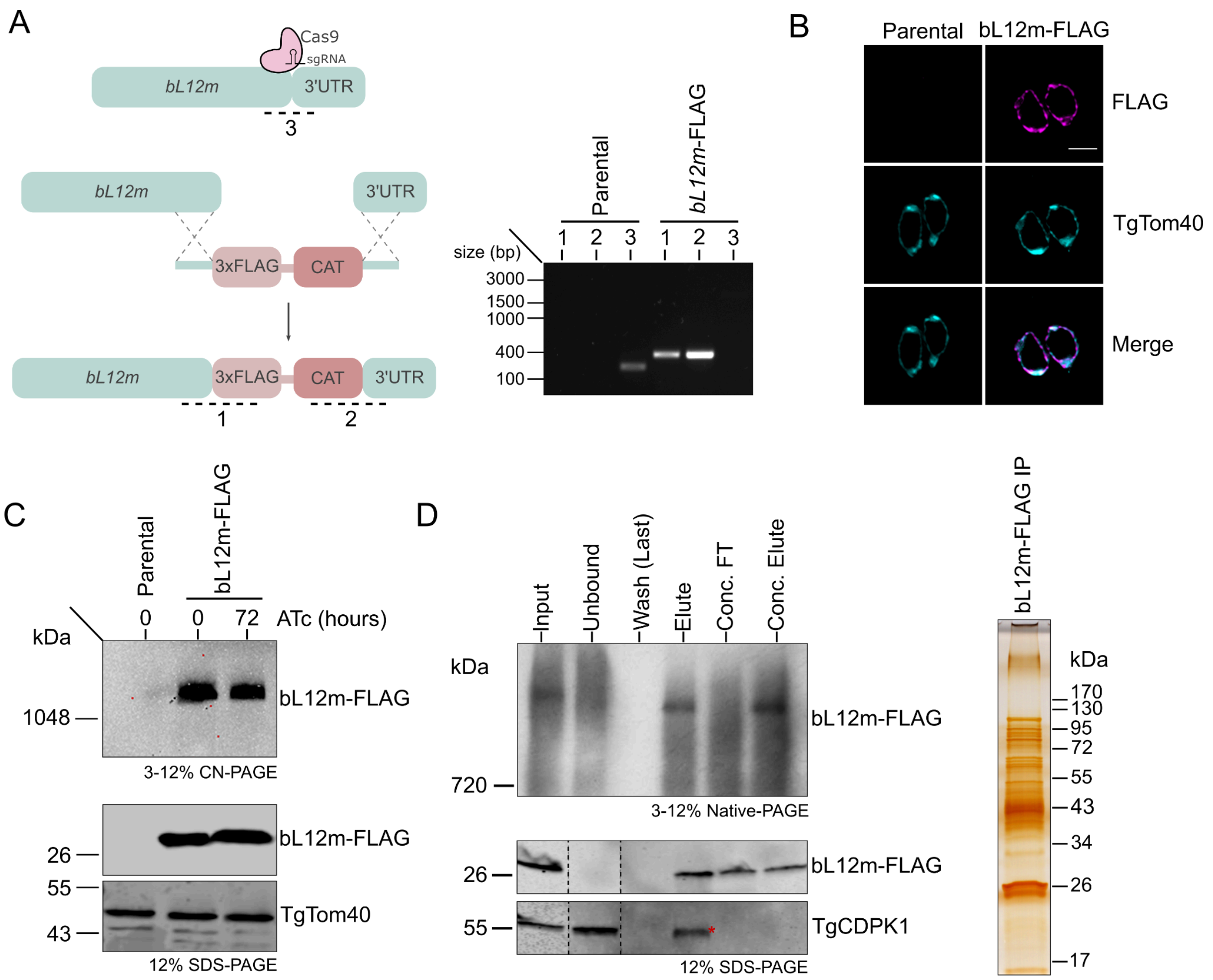

Supplementary Fig 1. Generating FLAG tagged bL12m cell line and subsequent immunoprecipitation (IP). A. (Left) schematic diagram depicting the strategy employed to generate endogenous C-terminal triple FLAG tagged bL12m cell line. (Right) PCR verification of successful endogenous tagging cassette insertion at the *bL12m* gene locus versus parental cell line. Expected PCR products labelled 1, 2 and 3 are indicated in the schematic on the left. CAT = Chloramphenicol Acetyltransferase; UTR = Untranslated region; sgRNA = single guide RNA. **B.** Immunostaining followed by fluorescence microscopy of intracellular *T. gondii* parasites with endogenous bL12m-FLAG probed with anti-FLAG (magenta) and anti-TgTom40 (cyan) antibodies. TgTom40 is a mitochondrial protein in *T. gondii*. Scale bar is 3 µM. **C.** Native and SDS-PAGE followed by western blot of *T. gondii* parasites showing migration of bL12m-FLAG in a high molecular weight complex, with endogenous TgTom40 showing equal loading for all samples. ATc treatment for 72 hours was done to show it does not disrupt mitoribosome formation. **D.** Native and SDS-PAGE followed by western blot of different fractions of bL12m-FLAG immunoprecipitation using anti-FLAG agarose beads; blots were probed with anti-FLAG and anti-TgCDPK1 antibodies as indicated. Discontinuous lanes are marked with dashed lines (Conc. = concentrated; FT = Flowthrough, * = IgH). TgCDPK1 is a cytosolic protein in *T. gondii*. Right panel, SDS-PAGE followed by silver staining of bL12m-Flag immunoprecipitated elute fraction. 20 µg of elute protein sample was loaded in the lane. Source data are provided as a Source Data file.

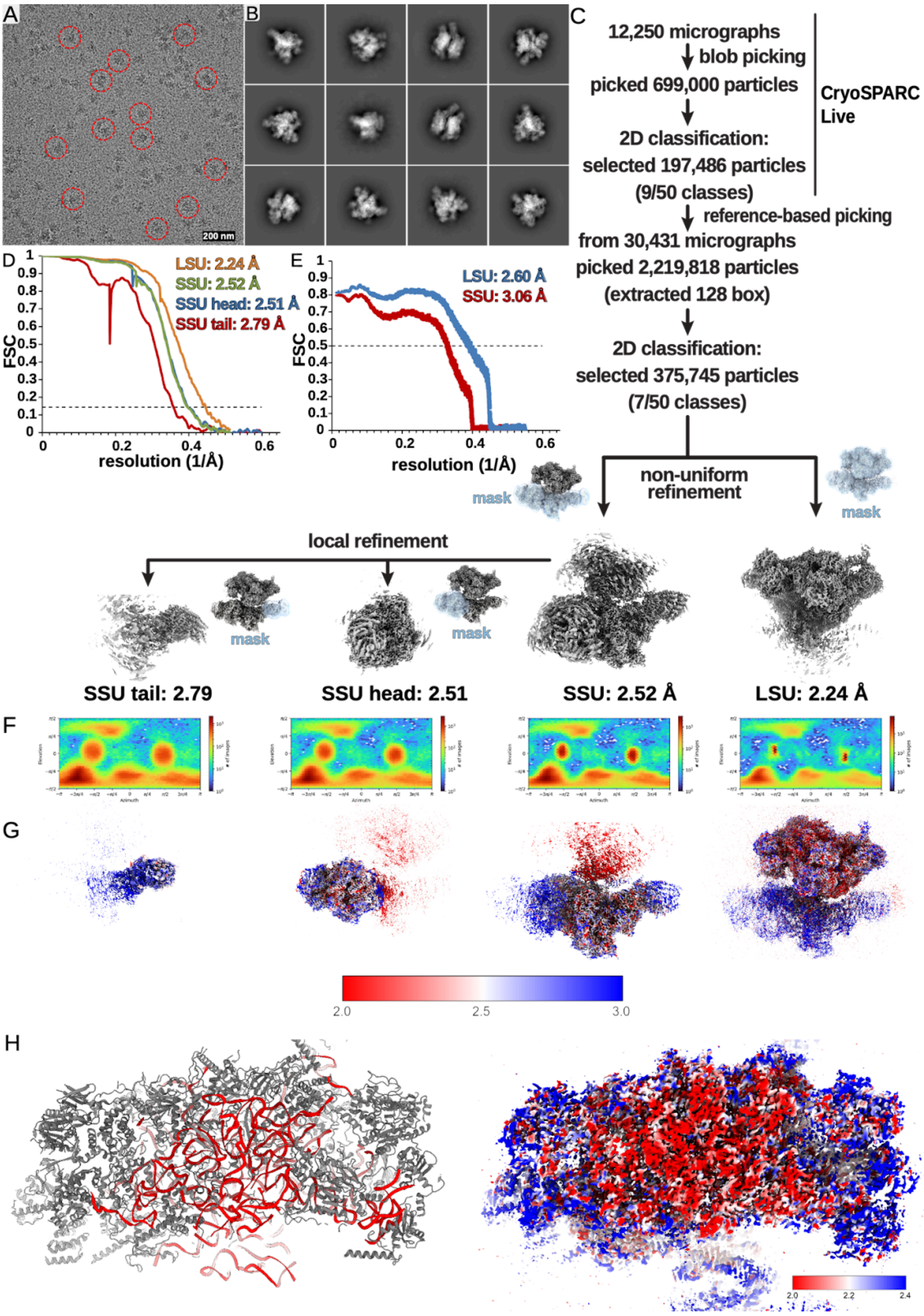
A
B
C
12,250 micrographs
blob picking
picked 699,000 particles
2D classification:
selected 197,486 particles
(9/50 classes)
CryoSPARC Live
reference-based picking
from 30,431 micrographs
picked 2,219,818 particles
(extracted 128 box)
2D classification:
selected 375,745 particles
(7/50 classes)
non-uniform refinement
mask
local refinement
D
LSU: 2.24 Å
SSU: 2.52 Å
SSU head: 2.51 Å
SSU tail: 2.79 Å
FSC
resolution (1/Å)
E
LSU: 2.60 Å
SSU: 3.06 Å
SSU tail: 2.79
SSU head: 2.51
SSU: 2.52 Å
LSU: 2.24 Å
F
G
H
200 nm

Supplementary Fig 2. Cryo-EM of the *T. gondii* mitochondrial ribosome. (A) Representative micrograph, **(B)** Representative 2D classes, **(C)** Data processing scheme, including reference generating for particle picking and the resulting maps for large subunit (LSU), small subunit (SSU) and two SSU submaps. 3D based classification was not able to separate any heterogeneity and further partition of the 2D classified particles was detrimental to the final resolution. **(D-E)** Resolution estimate by Fourier Shell Correlation (FSC) of half-maps (D) and model-map correlation for LSU and SSU models (E). (**F)** Viewing direction distribution plots indicating preferred orientation. **(G)** Local resolution estimates. **(H)** left LSU model (protein grey, RNA red) and right slice of LSU map with local resolution indicating that the RNA core is higher resolved than the surrounding protein subunits.

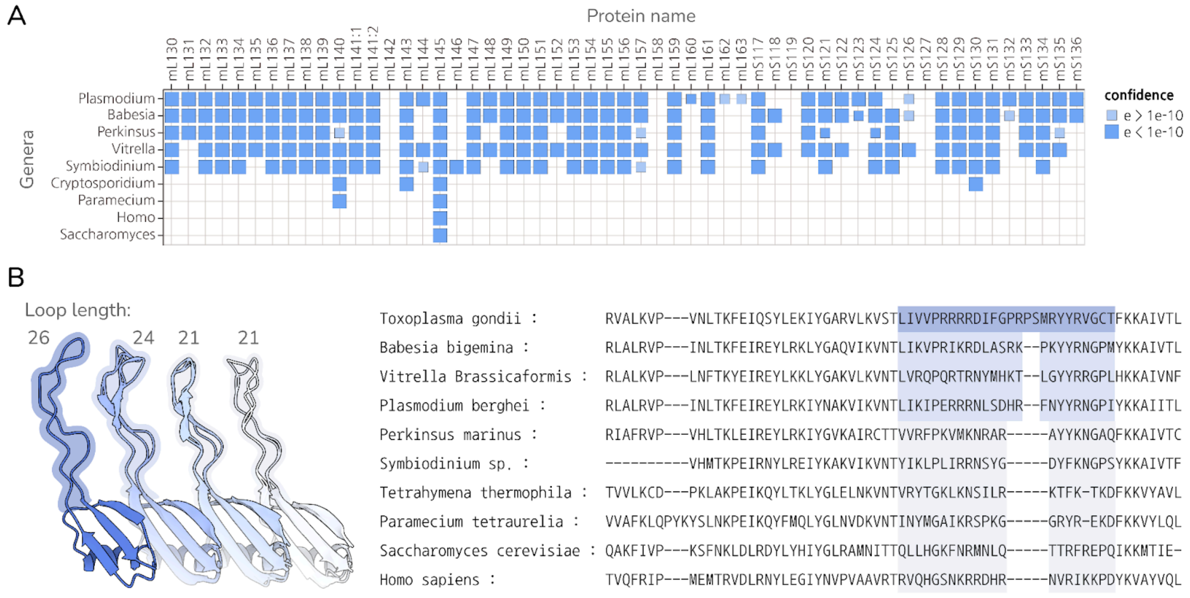

Supplementary Fig 3. Conservation of new mitoribosomal proteins across myzozoans or apicomplexans, and an apicomplexan structurally conserved insertion in uL23m. A. Schematic summary of PSI-Blast results obtained using the ToxoDB (https://toxodb.org/toxo/app/) annotation of each of the new mitoribosomal protein found in our structure (Supplementary Table 6). PSI-blast were run via NCBI, in two iterations, each time against a list of selected genera (*Plasmodium; Babesia; Cryptosporidium; Perkinsus; Vitrella; Symbiodinium; Paramecium; Saccharomyces; Homo sapiens*). **B.** Alignment and structural prediction of uL23m homologs from the same group of organisms as above, showing the apicomplexan 3-5 amino-acid insertion that encodes the structurally conserved loop identified within the *Toxoplasma* mitoribosome exit tunnel.

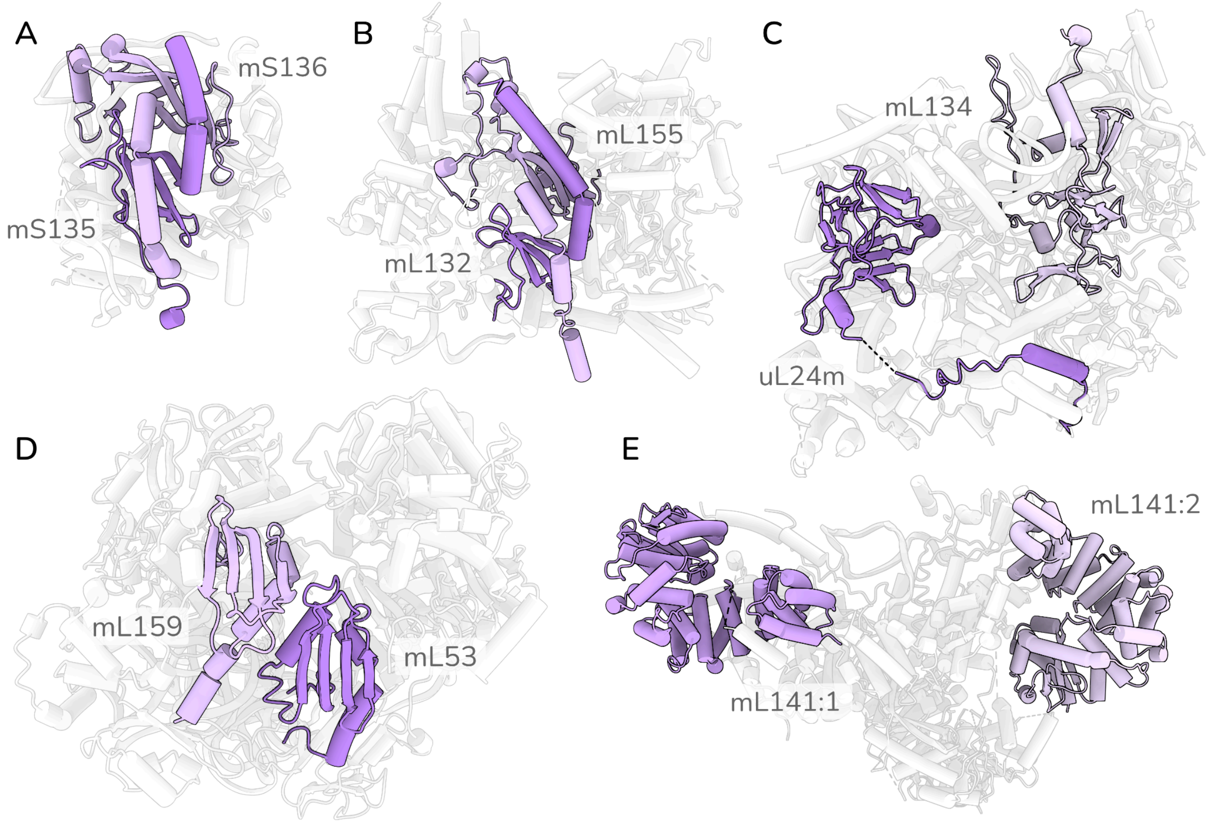

Supplementary Fig 4. "Twin-elements" present on the *T. gondii* mitoribosome. A. SSU bound PC4-like TF heterodimer binding nucleic acid. **B.** LSU bound PC4-like TF heterodimer. **C.** Incorporation of orthologous copy of uL24m, mL134. **D.** L7_L12 stalk base harbouring a mL159, with identical tertiary structure and sequence closely related to mL53, likely recent duplication. **E.** The two copies of mL141 on the mitoribosome.

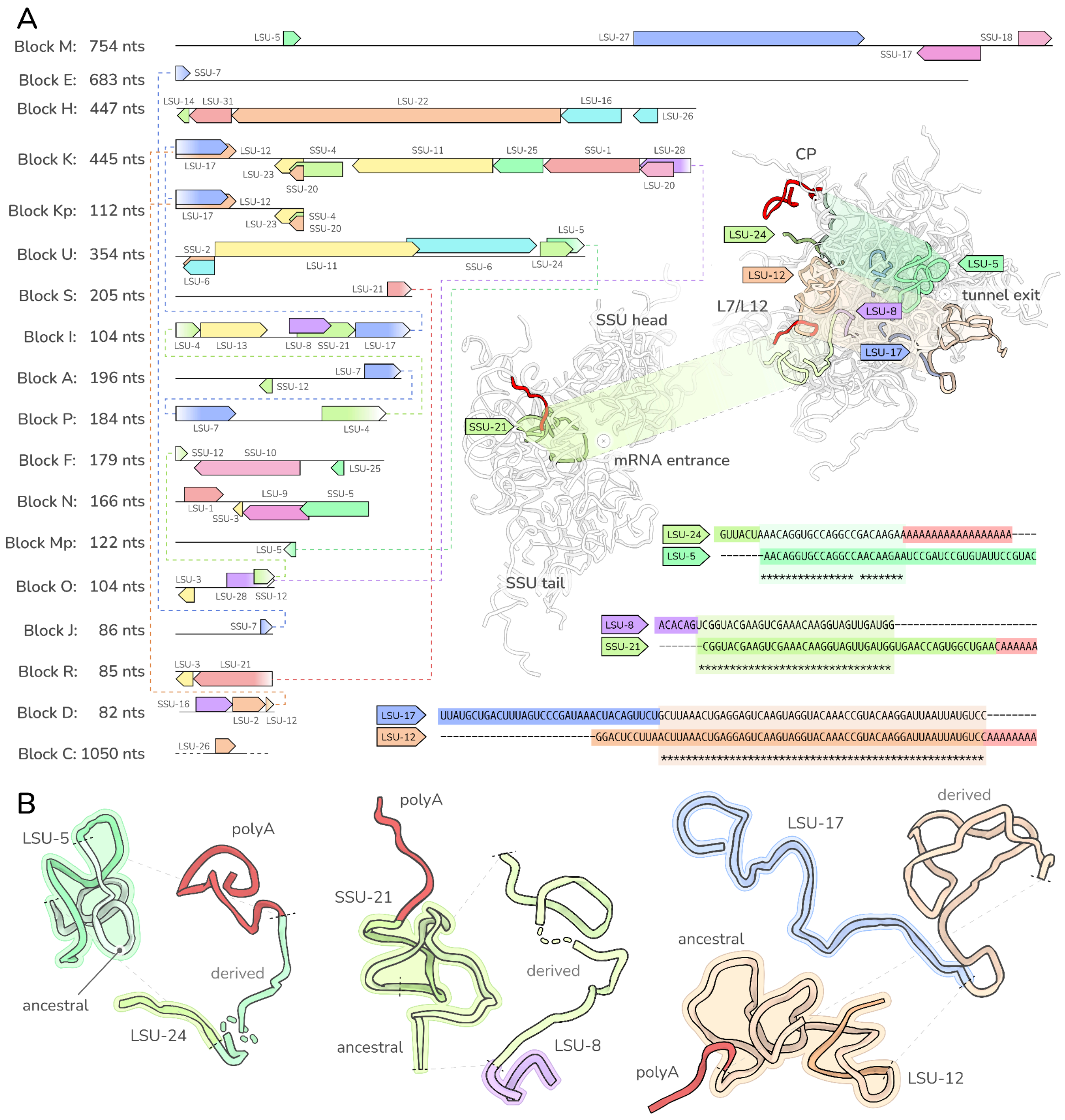

Supplementary Fig 5. Genomic architecture of rRNA fragments and sequence reuse. A. (left) schematic representation of *T. gondii* mitochondrial genome blocks ordered by size, lengths to scale[24,25]. Fragment locations are indicated, and fragments coloured sequentially. Fragments linked by coloured lines are split between multiple blocks and require these to be contiguous in the genomic arrangement to ensure complete fragment transcription. (right) 3D rRNA structure. Overlapping rRNA fragments highlighted together with associated sequence alignments shown below. **B.** Structural comparison of regions with overlapping rRNA sequence. Fragment regions with structural similarity to the bacterial ancestor labelled as ancestral. Region with shared sequence but without similarity to bacteria labelled as derived. Post transcriptional poly-A tails coloured red.

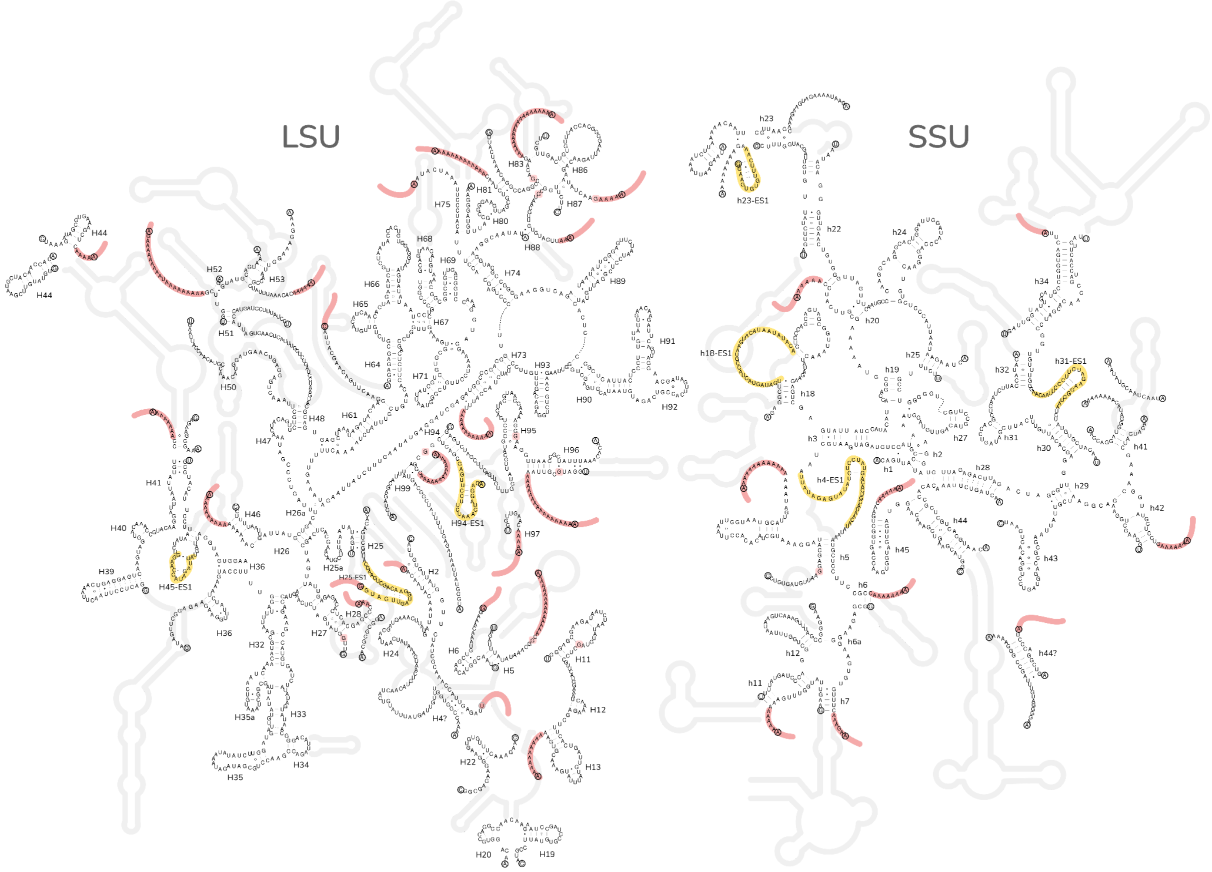

Supplementary Fig 6. 2D diagram of modelled *T. gondii* rRNA. Secondary structure diagram of modelled sequence shown overlaid on the closest representative structure from *T. thermophila*. Major conserved helices numbered following the bacterial nomenclature. Expansions and additional nucleic acid present in *T. gondii* highlighted in yellow. Post-transcriptional poly-A tails highlighted in red. Individual observations of A-G discrepancies highlighted in red.

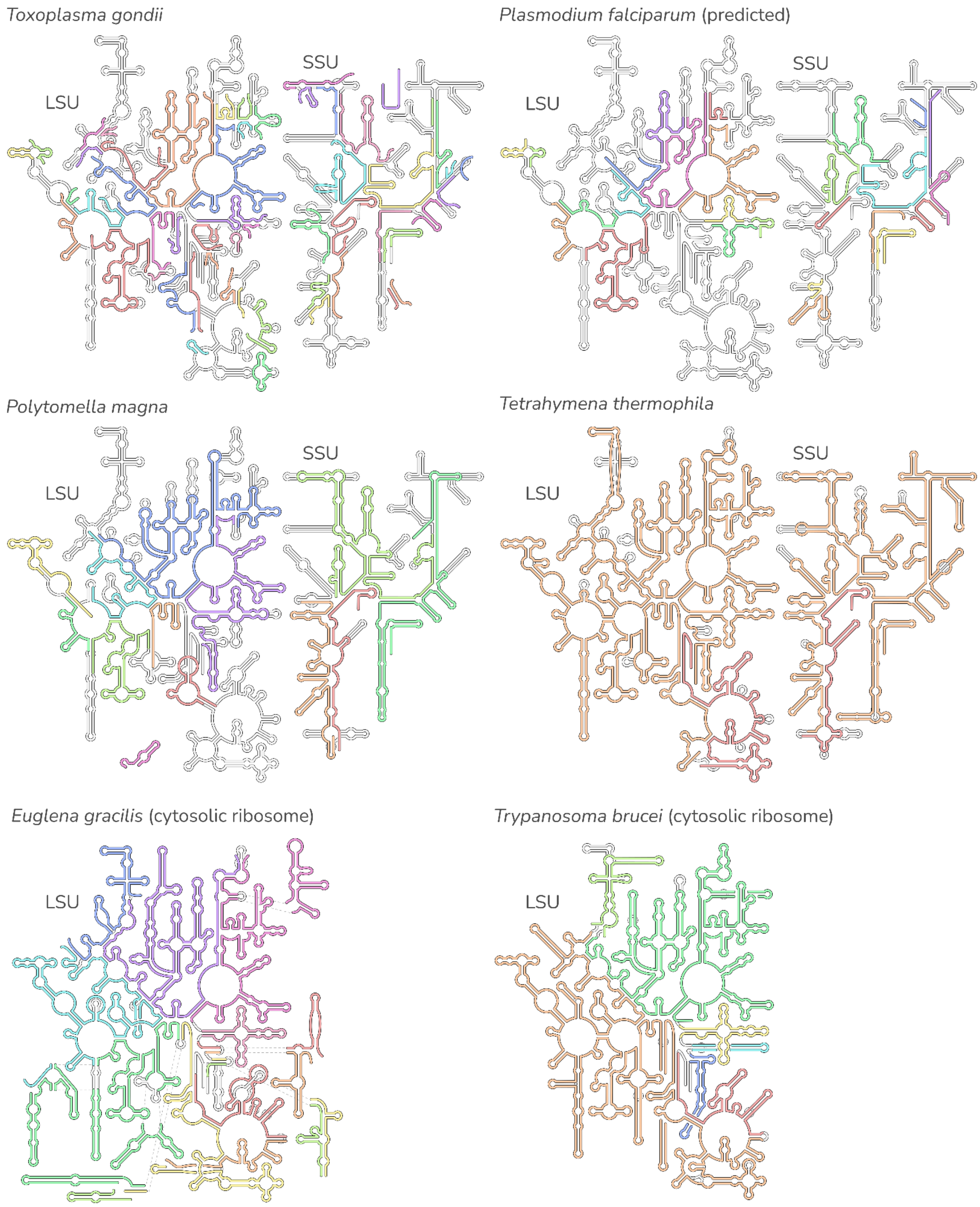

Supplementary Fig 7. rRNA fragmentation comparison. Comparison of the five fragmented ribosomal secondary structures together with the predicted secondary structure of *P. falciparum* highlighting fragmentation convergence. *T. gondii* fragmentation termini consistently coincide with those of the mitoribosome in *P. magna*, and *T. thermophila* as well as in the cytosolic ribosome of *T. brucei* and *E. gracilis* indicating the overall convergence of rRNA fragmentation. The small subunits for *T. brucei* and *E. gracilis* are not shown as they are unfragmented.

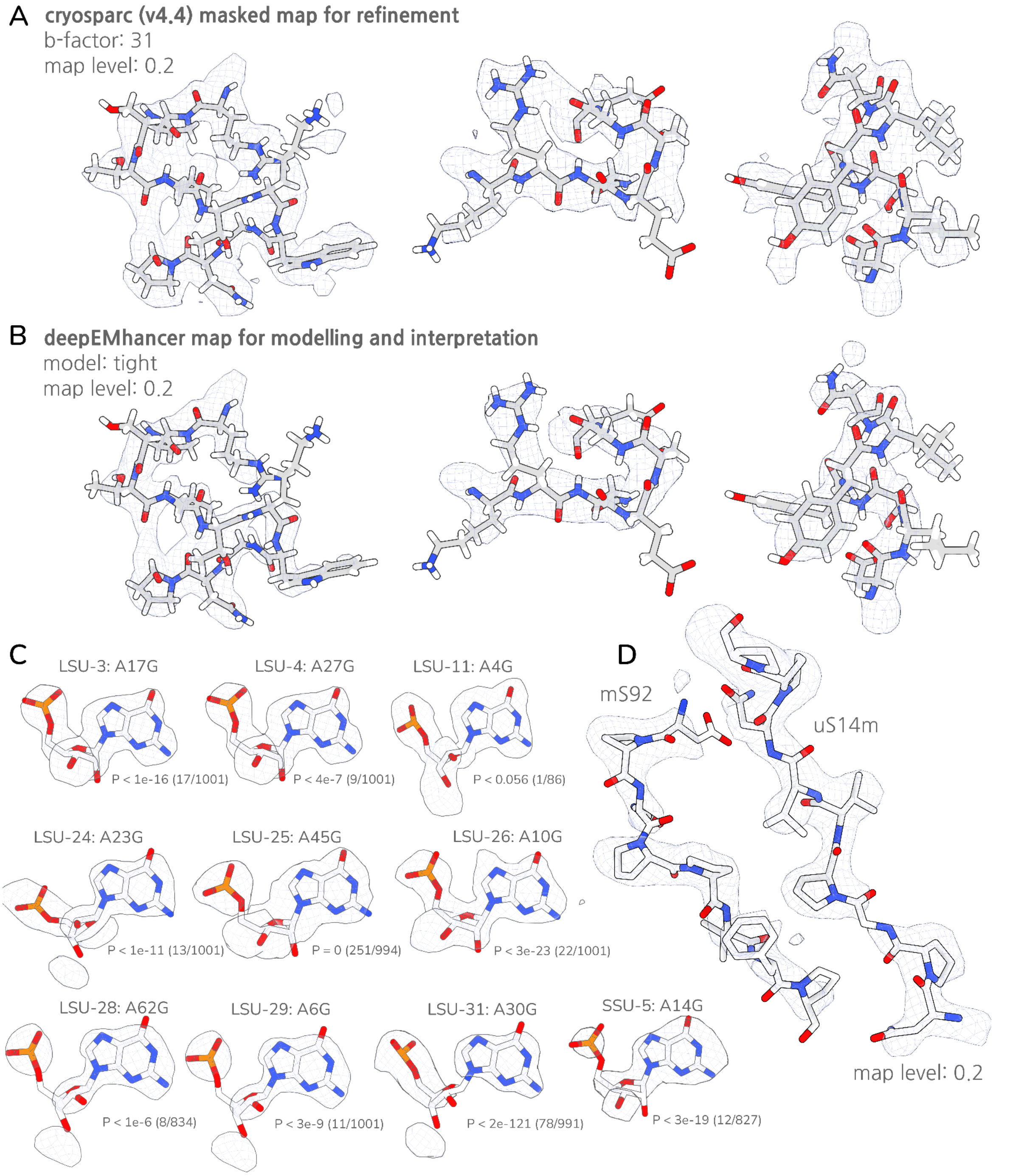

Supplementary Fig 8. CryoEM density examples. A-B. Examples drawn as an iso-surface at map level 0.2 for comparison of density from cryoSPARC masked refinement **(A)** before and **(B)** after enhancement by deepEMhancer using the "tight" model present. DeepEMhancer densities used for model building and interpretation, original cryoSPARC densities used for model refinement. **C.** Density examples for positions with observed A to G transitions from rRNA sequencing, bracketed numbers indicate unique transversions observed within a pool of 1000 analysed fragments containing reads. Density examples for positions with observed A to G transitions with calculated e-values from rRNA seq. Bracketed numbers indicate unique transversions observed within a pool of 1000 analysed fragment containing reads. **D.** Protein density examples for extensions of uS14m and mS92 covering previous position of uS3m.

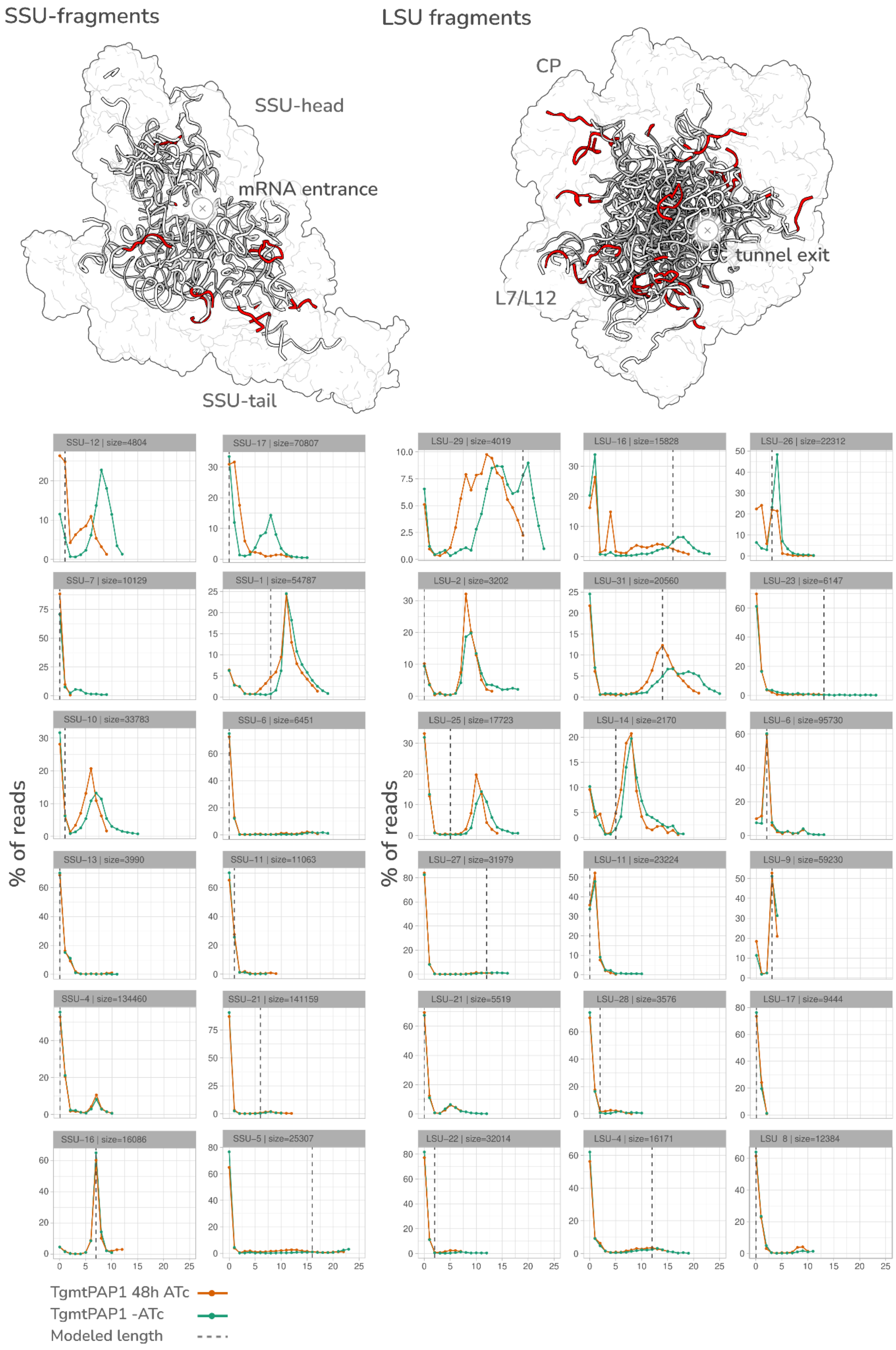

Supplementary Fig 9. Poly-A tails added to rRNAs are integrated into the mitoribosome RNA core. Top, distribution of poly-A tails (red) across the *T. gondii* mitoribosome. Bottom, analysis of rRNA abundance and ploy-A tail length in parental (-ATc, green) and TgmPAP1 depleted parasites (48h +ATc red).

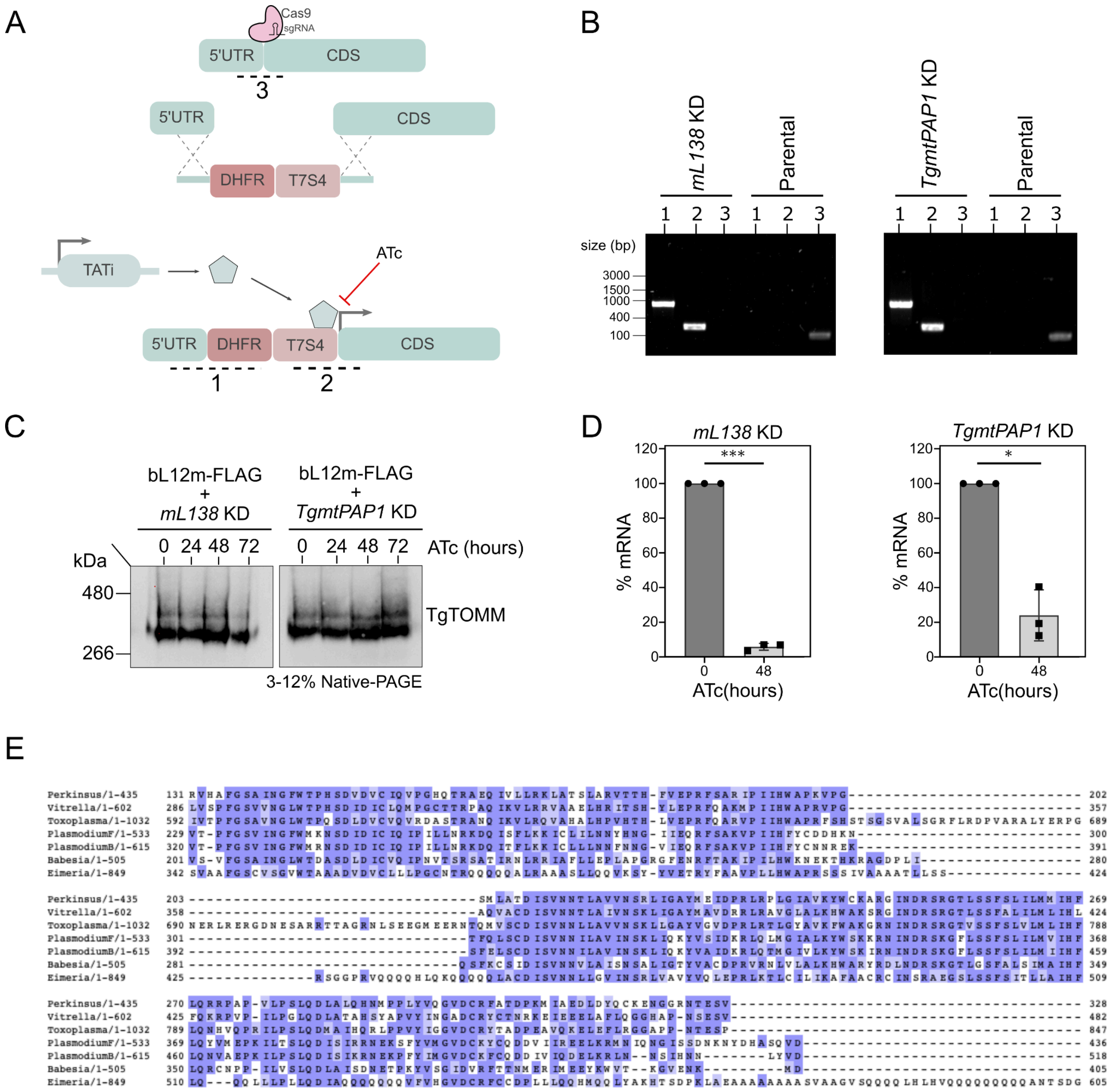

Supplementary Fig 10. Generation and validation of *mL138* and *TgmtPAP1* knockdown mutant. A. Schematic diagram depicting strategy employed to insert a regulatable promoter at *mL138* and *TgmtPAP1* 5'UTR locus, used to downregulate gene expression by addition of anhydrotetracycline (ATc). **B.** PCR verification of successful promoter replacement for *mL138* and *TgmtPAP1* versus in the parental cell line, using locus-specific primers. Expected PCR products labelled 1, 2 and 3 are represented in the schematic in (A). **C.** Native PAGE followed by western blot of *mL138* and *TgmtPAP1* knockdown cell lines in the background of bL12m-FLAG cell line, probed with TgTom40 antibody. (TOM = Translocase of the Outer Mitochondrial Membrane). **D.** qRT-PCR analysis of *mL138* and *TgmtPAP1* promoter replacement cell lines showing transcript level changes of respective genes in the parasite upon ATc treatment for 48 hours compared to no ATc treatment. Experiment was done in triplicates and single sample t test was performed to obtain the p-value for significance (***p = 0.0001, *p = 0.0122). **E.** *TgmtPAP1* was identified via BLAST search of the ToxoDB.org database using human mitochondrial poly-A polymerase (*MTPAP*) sequence as bait. Two hits were identified with high significance e-values (TGME49_257800, e-value 8e-12; TGGT1_281370, e-value 9e-14). Of those two TGGT1_281370 has evidence for

mitochondrial localization[26] and essentiality for growth[27] and thus focused on this protein for further analysis. Sequence alignment showing conservation of predicted polymerase domain of mitochondrial poly-A polymerase (TgmtPAP1) in various apicomplexans. Source data are provided as a Source Data file

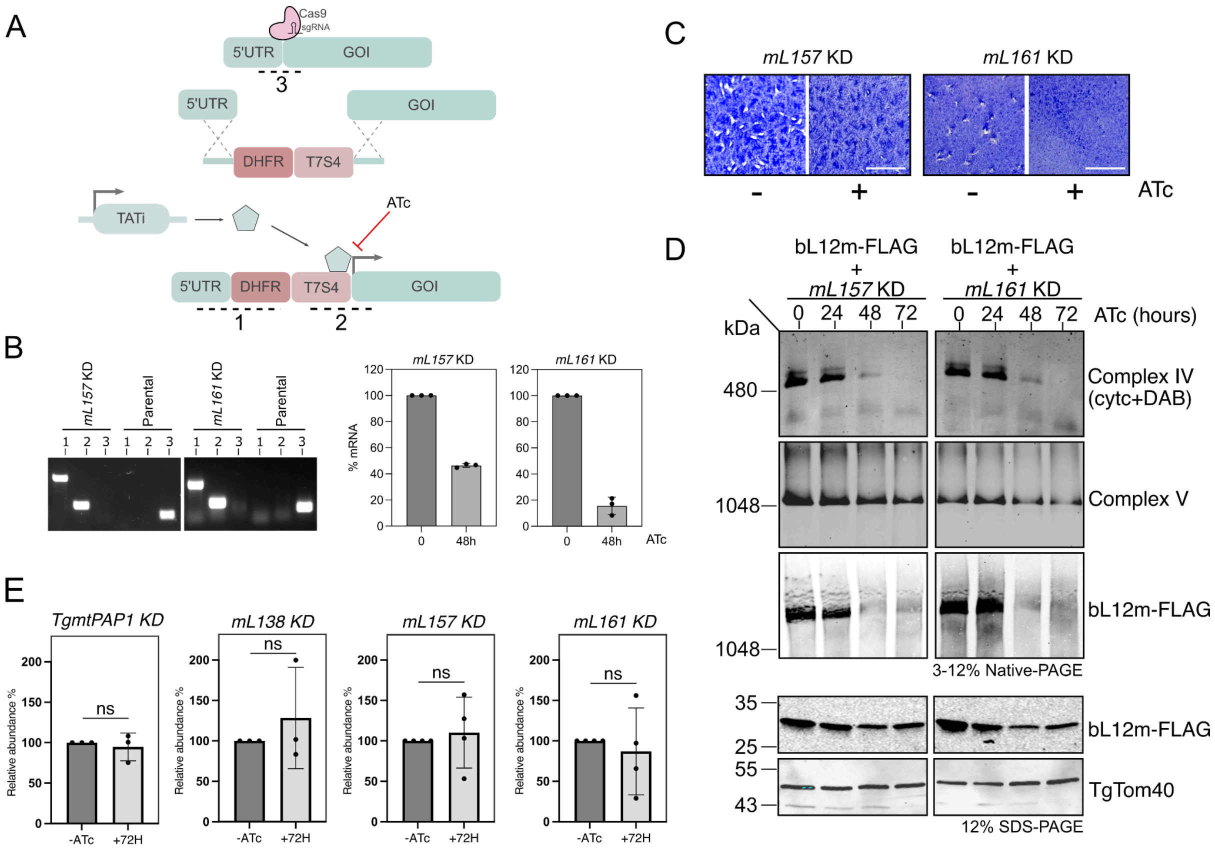

Supplementary Fig 11. **Generation, validation and mitoribosome defect upon *mL157* and *mL161* knockdown mutants. A.** Schematic diagram depicting strategy employed to insert a regulatable promoter at *mL157* and *mL161* 5'UTR locus, used to downregulate gene expression by addition of anhydrotetracycline (ATc). **B.** (left) PCR verification of successful promoter replacement for *mL157* and *mL161* versus in the parental cell line, using locus-specific primers. Expected PCR products labelled 1, 2 and 3 are represented in the schematic above. (right) qRT-PCR analysis of mL157 and TmL161 promoter replacement cell lines showing transcript level changes of the respective genes upon ATc treatment for 48 hours. Experiment was done in triplicates and single sample t test was performed to obtain the p-value for significance (***p = 0.0002, **p = 0.0021). **C.** Plaque assays assessing growth after 7 days of treatment. Scale bar = 1cm. **D.** Mitochondrial translation assay. Native-PAGE upper panel shows the mitochondrial translation dependent complex IV assembly and activity; middle panel shows the mitochondrial translation independent assembly of complex V. Lower panel shows mitoribosome formation assessed via Native-PAGE followed by immunoblotting against bL12m-FLAG using anti-FLAG antibody. Corresponding SDS-PAGE western blots are shown below the Native-PAGE panels depicting individual protein levels of bL12m-FLAG in whole cell sample. TgTom40 serves as a loading control. **E.** Quantification of complex V signal normalised to the signal from TgTom40 in three independent experiments performed with the lines shown in main Fig. 3 and in panel D here, demonstrate no change in complex V signal in those experiments. Two-tailed Welch's t-test was used. TgmtPAP1: n = 3; p = 0.6514; mL138 KD: n = 3; p = 0.5144; mL157 KD: n = 4; p = 0.6713; mL161 KD: n = 4; p = 0.6629; Source data are provided as a Source Data file.

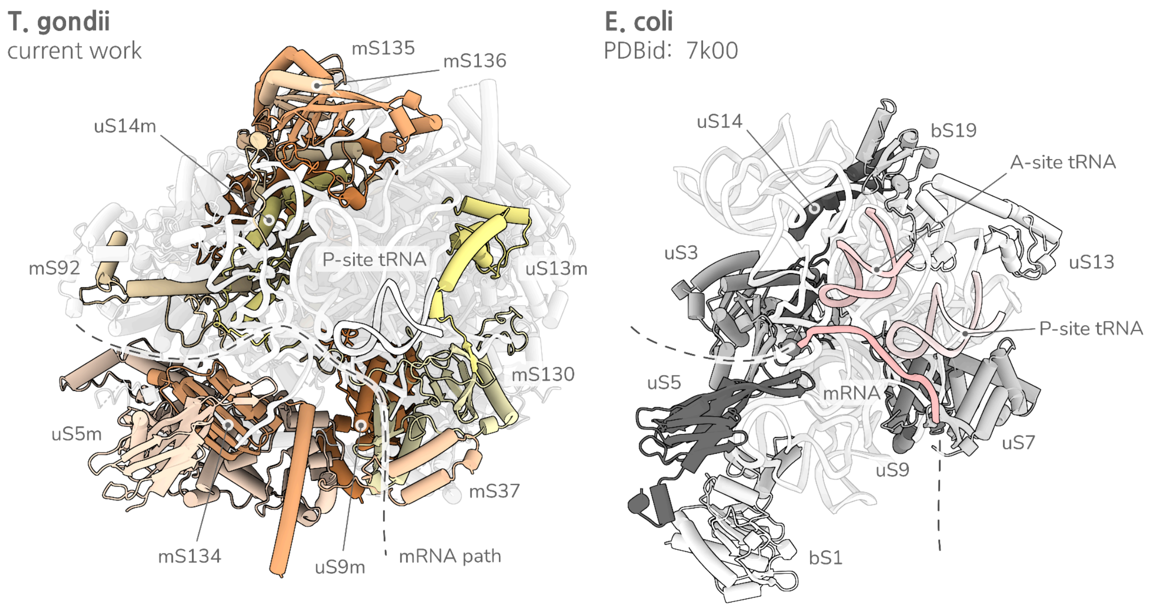

Supplementary Fig 12. Comparison between the mRNA channels in the Toxoplasma mitoribosome and the bacterial ribosome. Lateral view of mRNA binding path showing diverged protein architecture relative to bacterial ancestor. mRNA entrance to the left, exit to the right, highlighting presence of modelled t-RNAs in our current structure (left) and *E. coli* PDBid: 7K00 (right)[28]. Conserved bacterial and clade-specific mitoribosomal proteins coloured in shades of orange, bacterial ribosomal proteins in grey. mRNA and tRNAs in shades of red.

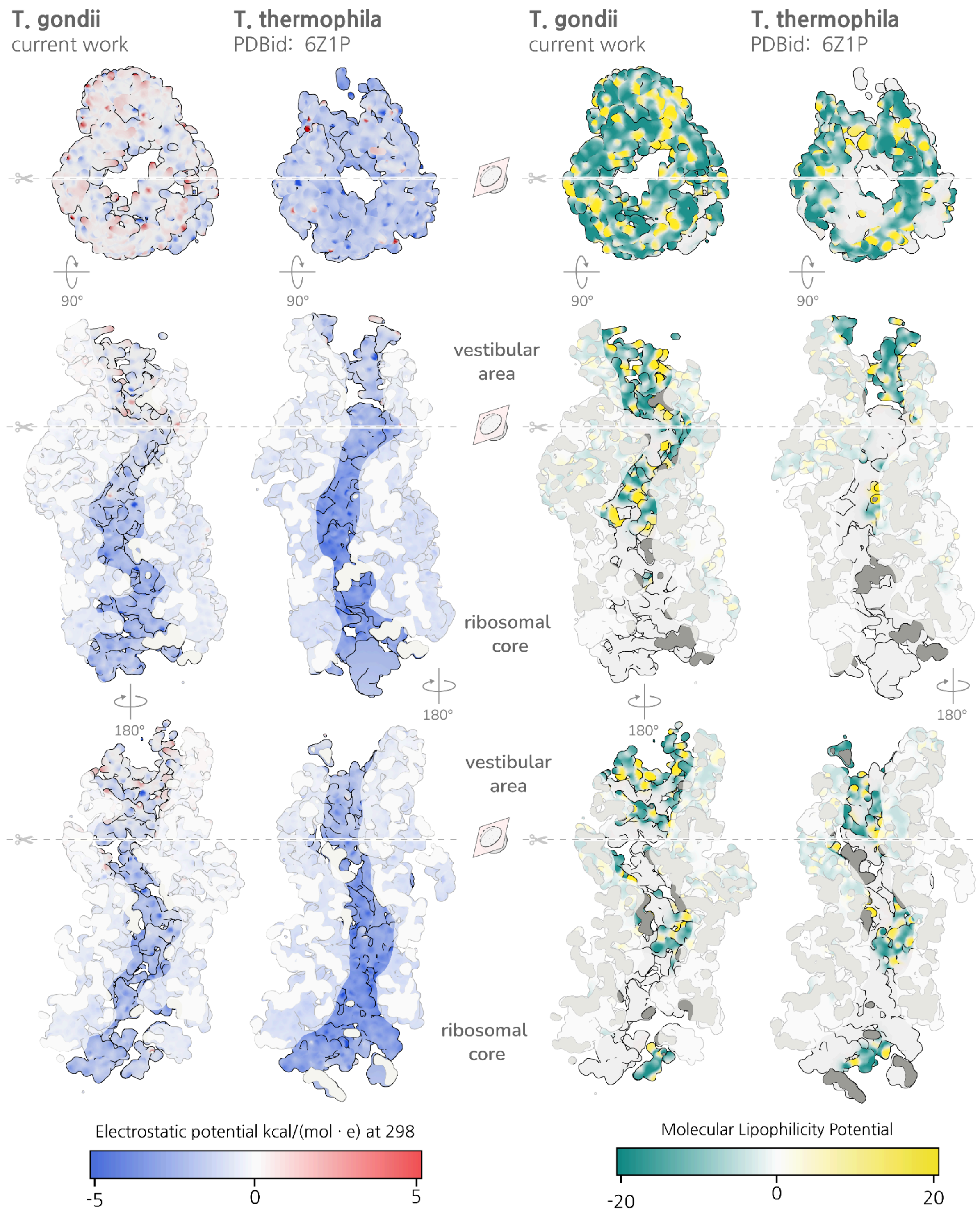

Supplementary Fig 13. Charge properties of the ribosomal exit tunnel. Transverse (top) and frontal (mid, bottom) slices of the ribosomal exit tunnel of our current structure (left) and *T. thermophila* PDBid: 6XWY (right) showing the lessened negative charge following loss of rRNA in our structure. Slice planes indicated by corresponding dashed lines. Tunnel shown as a surface representation drawn within 10Å of solvent accessibility. Coloured either by electrostatic potential estimated from partial atomic charges and Coulomb's law, or by Lipophilicity potential as estimated by pyMLP, both calculated in ChimeraX.

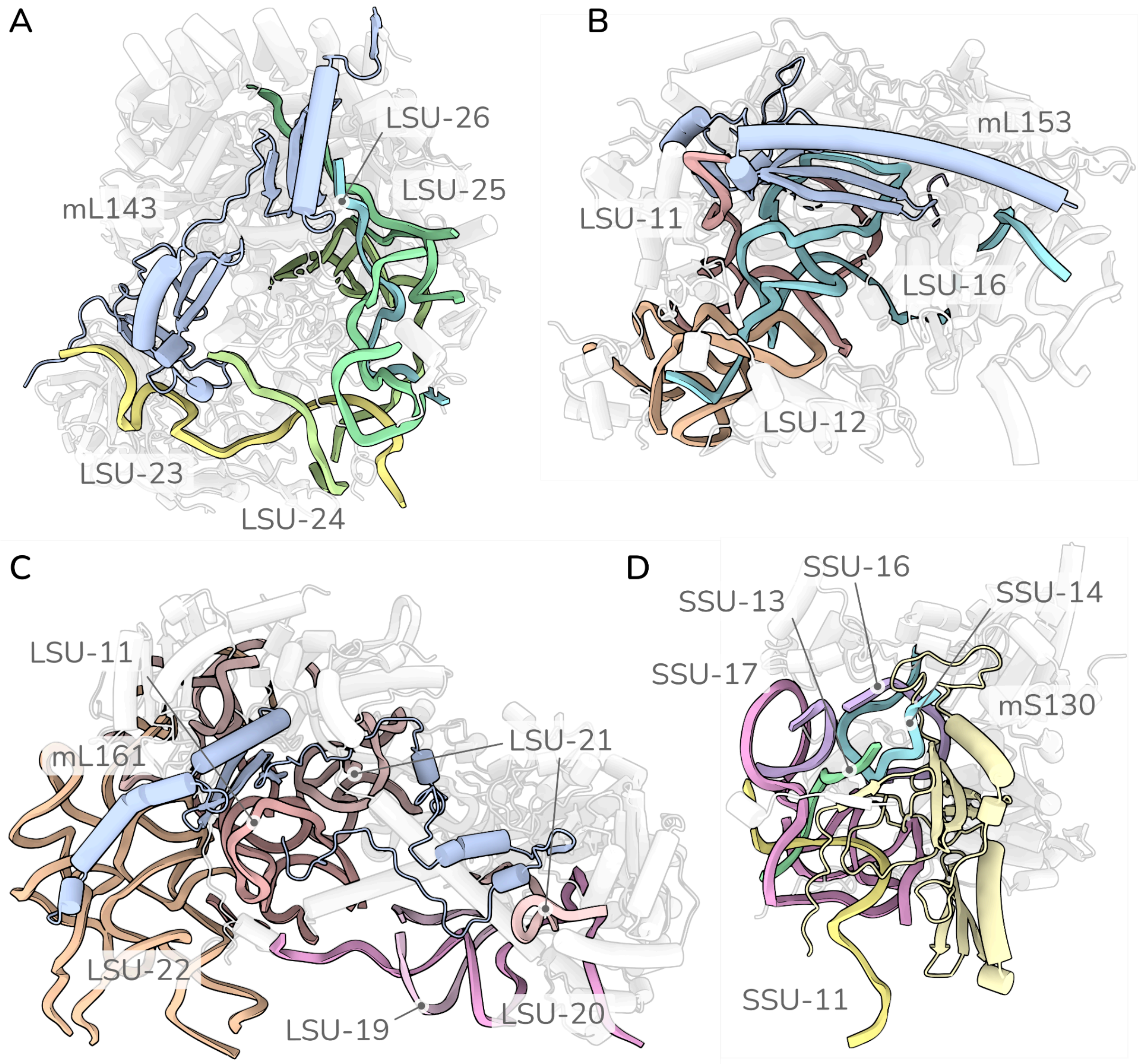

Supplementary Fig 14. Four ApiAP2 members form integral part of the *T. gondii* mitoribosome. A. tandem AP2 domain of mL143 bound to fragments LSU-24, 24, 25 and 26 in the central protuberance. **B.** Single AP2 domain containing mL153 bound to the apex of extended H45. **C.** Single AP2 domain containing mL161 bound by the subunit interface between fragments LSU-11 and LSU-22 with prominent terminal extensions. **D.** Tandem AP2 domain containing mS130 bound close to the SSU mRNA exit interacting with SSU-11, 13, 14, 16 and 17 through loop insertions and terminal extensions.
