# Supplementary material for: Numerous rRNA molecules form the apicomplexan mitoribosome via repurposed protein and RNA elements
Source: Nat Commun. 2025 Jan 18;16:817. doi: 10.1038/s41467-025-56057-9 (PMC11742926; doi:10.1038/s41467-025-56057-9)
Supplement: Supplementary file 2 — Description of Additional Supplementary Files [file 41467_2025_56057_MOESM2_ESM.pdf]

Description of Additional Supplementary Files

File Name: Supplementary Data 1
Description: Mass spectrometry results of bL12m-FLAG immunoprecipitation.

File Name: Supplementary Data 2
Description: Summary of the *T. gondii* mitoribosomal proteins identified in this work.

File Name: Supplementary Data 3
Description: Results of PSI-BLAST search for each of the clade-specific proteins.

File Name: Supplementary Data 4
Description: Summary of rRNA molecules identified from the density and references against the RNAseq. data from the bL12m-FLAG immunoprecipitation fraction.

File Name: Supplementary Data 5
Description: Sequences of short peptides and the corresponding geneID or genomic location.

File Name: Supplementary Data 6
Description: Summary of oligos used in the study.
