# Supplementary material for: Numerous rRNA molecules form the apicomplexan mitoribosome via repurposed protein and RNA elements
Source: Nat Commun. 2025 Jan 18;16:817. doi: 10.1038/s41467-025-56057-9 (PMC11742926; doi:10.1038/s41467-025-56057-9)
Supplement: Supplementary file 9 — Reporting Summary [file 41467_2025_56057_MOESM9_ESM.pdf]

nature portfolio

Corresponding author(s): Lilach Sheiner

Last updated by author(s): YYYY-MM-DD

Reporting Summary

Nature Portfolio wishes to improve the reproducibility of the work that we publish. This form provides structure for consistency and transparency in reporting. For further information on Nature Portfolio policies, see our Editorial Policies and the Editorial Policy Checklist.

Statistics

For all statistical analyses, confirm that the following items are present in the figure legend, table legend, main text, or Methods section.

n/a	Confirmed	
☐	☒	The exact sample size (*n*) for each experimental group/condition, given as a discrete number and unit of measurement
☐	☒	A statement on whether measurements were taken from distinct samples or whether the same sample was measured repeatedly
☐	☒	The statistical test(s) used AND whether they are one- or two-sided<br>*Only common tests should be described solely by name; describe more complex techniques in the Methods section.*
☒	☐	A description of all covariates tested
☐	☒	A description of any assumptions or corrections, such as tests of normality and adjustment for multiple comparisons
☐	☒	A full description of the statistical parameters including central tendency (e.g. means) or other basic estimates (e.g. regression coefficient) AND variation (e.g. standard deviation) or associated estimates of uncertainty (e.g. confidence intervals)
☐	☒	For null hypothesis testing, the test statistic (e.g. *F*, *t*, *r*) with confidence intervals, effect sizes, degrees of freedom and *P* value noted<br>*Give P values as exact values whenever suitable.*
☒	☐	For Bayesian analysis, information on the choice of priors and Markov chain Monte Carlo settings
☒	☐	For hierarchical and complex designs, identification of the appropriate level for tests and full reporting of outcomes
☒	☐	Estimates of effect sizes (e.g. Cohen's *d*, Pearson's *r*), indicating how they were calculated

Our web collection on statistics for biologists contains articles on many of the points above.

Software and code

Policy information about availability of computer code

Data collection	Microscopy: SoftWoRx
Data analysis	FIJI (v1.5.2)<br>Prism (v9)<br>Coot: 0.9.5<br>ChimeraX: 1.7rc202311290355 (2023-11-29)

For manuscripts utilizing custom algorithms or software that are central to the research but not yet described in published literature, software must be made available to editors and reviewers. We strongly encourage code deposition in a community repository (e.g. GitHub). See the Nature Portfolio guidelines for submitting code & software for further information.

Data

Policy information about availability of data

All manuscripts must include a data availability statement. This statement should provide the following information, where applicable:

- Accession codes, unique identifiers, or web links for publicly available datasets
- A description of any restrictions on data availability
- For clinical datasets or third party data, please ensure that the statement adheres to our policy

RNA-seq data: European Nucleotide Archive (PRJEB72258)

Research involving human participants, their data, or biological material

Policy information about studies with human participants or human data. See also policy information about sex, gender (identity/presentation), and sexual orientation and race, ethnicity and racism.

Reporting on sex and gender	NA
Reporting on race, ethnicity, or other socially relevant groupings	NA
Population characteristics	NA
Recruitment	NA
Ethics oversight	NA

Note that full information on the approval of the study protocol must also be provided in the manuscript.

Field-specific reporting

Please select the one below that is the best fit for your research. If you are not sure, read the appropriate sections before making your selection.

☒ Life sciences ☐ Behavioural & social sciences ☐ Ecological, evolutionary & environmental sciences

For a reference copy of the document with all sections, see nature.com/documents/nr-reporting-summary-flat.pdf

Life sciences study design

All studies must disclose on these points even when the disclosure is negative.

Sample size	For cryo-EM analysis sample sizes were determined based on our earlier works and experience, sufficient to obtain reliable results, as confirmed by the estimated resolution.<br>For all other experiments, sample size was not predetermined, all experiments were repeated as indicated. Samples sizes are consistent with previously published studies.
Data exclusions	For cryo-EM structure determination, particles that did not correspond to mitochondrial ribosomes were discarded by classification. For all other experiments, no exclusion criteria were included and no data was excluded.
Replication	All experiments were repeated multiple times independently. Variability between replicates was reported. All experiments have been reported where they lead to the statistical values were shown.
Randomization	Cryo-EM map resolution estimates by Fourier Shell Correclation were performed using half-maps from random half-sets.
Blinding	Investigators were not blinded during data acquisition or analysis

Reporting for specific materials, systems and methods

We require information from authors about some types of materials, experimental systems and methods used in many studies. Here, indicate whether each material, system or method listed is relevant to your study. If you are not sure if a list item applies to your research, read the appropriate section before selecting a response.

Materials & experimental systems

n/a	Involved in the study	
☐	☒	Antibodies
☐	☒	Eukaryotic cell lines
☒	☐	Palaeontology and archaeology
☒	☐	Animals and other organisms
☒	☐	Clinical data
☒	☐	Dual use research of concern
☒	☐	Plants

Methods

n/a	Involved in the study	
☒	☐	ChIP-seq
☒	☐	Flow cytometry
☒	☐	MRI-based neuroimaging

Antibodies

Antibodies used	Primary antibodies:<br>Mouse Anti-FLAG (Merck, F3165 Flag M2)<br>Rabbit anti-TgTom40<br>Guinea-Pig anti-CDPK1<br><br>Secondary antibodies:<br>anti-mouse IRDye 800CW (Li-COR 926-32210)<br>anti-rabbit IRDye 680RD (Li-COR 926-68071)<br>Alexa Fluor Goat anti-Rabbit 488 (A-11034)<br>Alexa Fluor Goat anti-Mouse 594 (A-11032)<br>anti-Guinea-Pig 800CW (Li-COR 926-32411)
Validation	Commercial antobodies were validated as described on manufacturer's website.<br>Rabbit anti-TgTom40 (van Dooren et al., 2016)<br>Guinea-Pig anti-CDPK1 (Lourido et al., 2010)

Eukaryotic cell lines

Policy information about cell lines and Sex and Gender in Research

Cell line source(s)	HFF-1 (ATCC SCRC-1041)<br>RHTATiΔku80 Sheiner et al (2011) "A systematic screen to discover and analyze apicoplast proteins identifies a conserved and essential protein import factor"<br>VERO ATCC CCL-81
Authentication	HFF cells were authenticated by ATCC using intraspecific STR analysis. Toxoplasma cell lines were validated by PCR where appropriate.
Mycoplasma contamination	Testing for mycoplasma was not performed.
Commonly misidentified lines<br>(See ICLAC register)	No commonly mis-identified cell lines were used in this study

Plants

Seed stocks	NA
Novel plant genotypes	NA
Authentication	NA
